# Novel Utilization of Circulating Tumor DNA in Primary Dedifferentiated Seminal Vesicle Adenocarcinoma: A Case Report of Molecular Clearance Following Multimodal Therapy

**DOI:** 10.14740/jmc5238

**Published:** 2026-01-13

**Authors:** Kamil Malshy, Brendan J. Guercio, Laena Hines, Sarah K. Findeis, Philip A. Sutera, Shawn W. Thomas, Jean V. Joseph

**Affiliations:** a Department of Urology, University of Rochester Medical Center, Rochester, NY, USA; b James P. Wilmot Cancer Institute, University of Rochester, Rochester, NY, USA; c Department of Pathology and Laboratory Medicine, University of Rochester Medical Center, Rochester, NY, USA; d Department of Radiation Oncology, University of Rochester Medical Center, Rochester, NY, USA; e Florida Urology Center, Ormond Beach, FL, USA

**Keywords:** Adenocarcinoma, Seminal vesicles, Biomarker, ctDNA, Prostate

## Abstract

Primary seminal vesicle adenocarcinoma (PSVA) is an exceptionally rare malignancy, with fewer than 100 cases reported worldwide and poses significant diagnostic and surveillance challenges due to its deep pelvic location, nonspecific clinical manifestations, frequent coexistence with other genitourinary malignancies, and lack of validated serum tumor markers. A 77-year-old male with long-standing lower urinary tract symptoms and mildly elevated prostate-specific antigen was found to have a large (7.4 cm) predominantly cystic pelvic mass replacing the left seminal vesicle on magnetic resonance imaging. Histologic evaluation revealed synchronous high-grade prostate adenocarcinoma and a distinct dedifferentiated carcinoma not arising from prostatic tissue. Comprehensive immunohistochemical analysis supported a diagnosis of PSVA. The patient underwent robotic-assisted radical prostatectomy with *en bloc* excision of the seminal vesicle mass, rectal repair, and ureteral reimplantation. Postoperatively, prostate-specific antigen remained undetectable; however, tumor-informed circulating tumor DNA (ctDNA) testing detected molecular residual disease. Following completion of radiotherapy, ctDNA became undetectable, and the patient has remained disease-free at nearly 1 year of follow-up. This case highlights the importance of comprehensive imaging, detailed immunohistochemical profiling, and aggressive multimodal management in PSVA, and represents the first documented report of molecular clearance using ctDNA after treatment for this rare malignancy. While causal inference cannot be established from a single case, this report suggests that ctDNA may serve as a promising adjunctive tool for postoperative surveillance in rare urologic cancers lacking reliable serum biomarkers.

## Introduction

Primary seminal vesicle adenocarcinoma (PSVA) is an exceptionally uncommon malignancy, with fewer than 100 documented cases worldwide [1]. In most clinical scenarios, the seminal vesicles (SVs) are involved secondarily, most often through direct extension from locally advanced prostatic adenocarcinoma (stage T3b) or, less frequently, from bladder or rectal cancers [2]. True primary tumors of this site represent only a small fraction of all SV lesions.

PSVA most often presents in men in their sixth or seventh decade of life, with a median reported tumor size of 5–6 cm at diagnosis [3]. Owing to its deep pelvic location and typically indolent early course, the disease frequently remains asymptomatic until it has reached an advanced stage. Presentations can include lower urinary tract symptoms (LUTS), hematospermia [4], pelvic or perineal discomfort, hematuria, or occasionally obstructive uropathy [3, 5, 6]. Multiparametric magnetic resonance imaging (MRI) is valuable for defining tumor location and local extension, but histologic evaluation with immunohistochemistry remains essential for a definitive diagnosis [5]. The overall prognosis is unfavorable in advanced disease [3], although rare durable control is possible with aggressive multimodal treatment strategies [3, 7].

At presentation, prostate-specific antigen (PSA) levels are frequently within the normal range [3], which can delay comprehensive diagnostic evaluation and lead to management focused on symptom relief rather than definitive investigation. Following treatment, the lack of consistently reliable serum tumor markers, most commonly cancer antigen 125 (CA-125) [8], with less consistent reports involving carcinoembryonic antigen (CEA) [9], further complicates surveillance. These diagnostic and follow-up challenges highlight the potential role of circulating tumor DNA (ctDNA) as an adjunctive monitoring modality. ctDNA testing has demonstrated value in detecting minimal residual disease and early recurrence across several solid tumors [10, 11], and may be particularly advantageous in PSVA, where standard markers offer little guidance.

Here, we report a rare case of dedifferentiated PSVA occurring synchronously with prostate adenocarcinoma, managed with robotic-assisted pelvic resection, rectal repair, ureteral reimplantation, and adjuvant radiotherapy. To our knowledge, this is the first documented case to demonstrate complete molecular clearance using ctDNA after multimodal therapy for PSVA.

## Case Report

A 77-year-old man presented with a several-year history of moderate to severe mixed LUTS, managed with tamsulosin and tadalafil. PSA levels ranged between 3 and 7 ng/mL over this period, with intermittent use of 5-alpha-reductase inhibitors, which may have masked higher true PSA values. Pelvic MRI performed in February 2021 demonstrated a Prostate Imaging–Reporting and Data System (PI-RADS) 4 lesion in the transition zone without suspicious peripheral zone findings; the prostate volume was 70 mL. Subsequent cognitive prostate needle biopsy revealed benign prostatic hyperplasia, and management at that time remained focused on symptom control.

**Figure 1:**
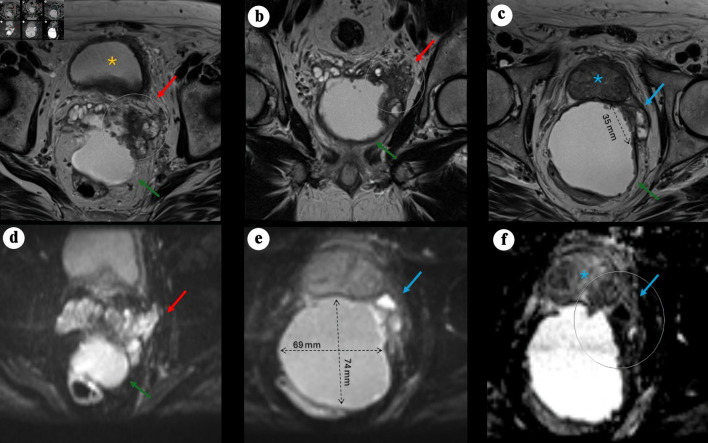
Pelvic MRI demonstrating a large cystic pelvic mass with features suggestive of malignancy. (a) Axial T2-weighted image showing a large, predominantly cystic mass (green arrow) situated between the rectum and the posterior left peripheral zone of the bladder (yellow asterisk), associated with a severely distorted left SV (red arrow) demonstrating hypoattenuation, suspected to originate from a left SV mass, with rim enhancement and peripheral nodularity (arrow). (b) Coronal view showing the mass located posteriorly and extending caudally. (c) Axial view demonstrating an additional smaller cystic focus (3.6 × 1.4 cm) between the left posterolateral mid-gland and the main lesion. Irregular T2-hypointense tissue extends between the left inferior SV, the cystic mass, and the left posterolateral base of the prostate, suspicious for malignancy (PI-RADS 5) with extraprostatic extension. (d) Axial DWI view of the mass. (e) Axial DWI showing marked diffusion restriction within the prostatic lesion and along the cyst lining (69 × 74 mm process). (f) Axial apparent diffusion coefficient (ADC) map demonstrating corresponding low signal. MRI: magnetic resonance imaging; PI-RADS: Prostate Imaging–Reporting and Data System; SV: seminal vesicle; DWI: diffusion-weighted imaging.

In April 2024, repeat pelvic MRI (Fig. 1) revealed a large (7.4 × 6.8 × 6.7 cm) predominantly cystic mass between the rectum and the posterior peripheral zone, with peripheral rim enhancement and diffusion-restricting nodularity. An additional smaller cystic focus (3.6 × 1.4 cm) was seen between the left posterior lateral mid-gland and the main lesion. Irregular T2-hypointense tissue extended between the left inferior SV, the cystic mass, and the left posterior lateral base of the prostate, suspicious for malignancy (PI-RADS 5) with extra prostatic extension. The transition zone was mildly enlarged without suspicious lesions. No abnormal pelvic lymph nodes or bone lesions were identified, and sigmoid diverticulosis was incidentally noted.

In the following month, systematic and targeted prostate biopsy revealed Gleason 3 + 5 = 8 (grade group 4) adenocarcinoma in one core and Gleason 4 + 3 = 7 (grade group 3) adenocarcinoma in two cores. Aspiration cytology from the periprostatic cystic lesion demonstrated malignant cells with an immunohistochemical profile most consistent with a poorly differentiated carcinoma, with features suggestive of a possible urothelial or squamous cell origin (nonspecific immune profile per outside report; however, cytokeratin (CK)5/6 and AE1/3 were positive and CK7 was focally positive). Significant LUTS improvement was noted following the aspiration. Colonoscopy and flexible sigmoidoscopy found no evidence of a rectal or colonic primary tumor or fistula.

**Figure 2:**
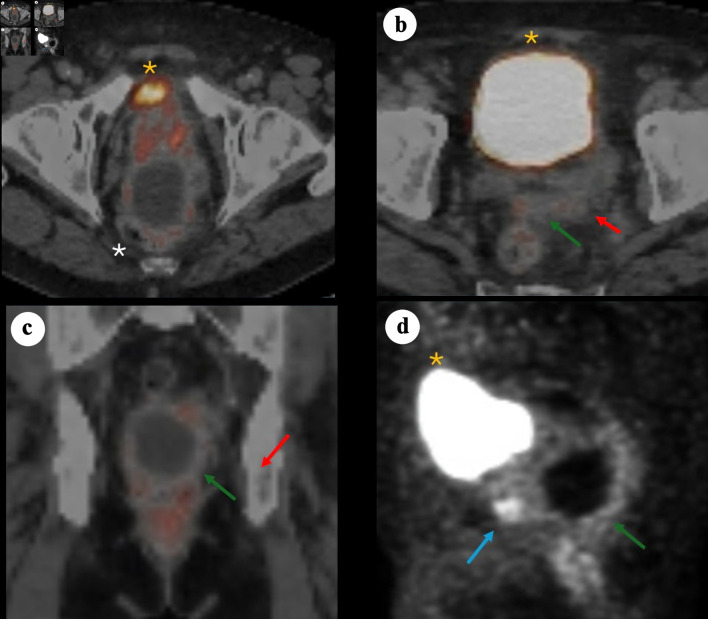
68Ga-PSMA PET/CT images demonstrating a large cystic mass in the pelvis consistent with primary SV adenocarcinoma. (a) Axial PET/CT showing bladder anteriorly (yellow asterisk), prostate (minimal PSMA uptake) and adjacent cystic pelvic mass (white asterisk). (b) Axial PET/CT showing bladder anteriorly (yellow asterisk), with posterior a solid component (red arrow) arousing from the left SV and contiguous tissue extension (green arrow). (c) Coronal fused PET/CT showing the relationship between the cystic mass and the adjacent prostate/SV region, with the solid enhancing component (red arrow) and soft tissue extension (green arrow). (d) Sagittal PET maximum intensity projection illustrating the prostate solid lesion component (blue arrow) and cyst (green arrow) posterior and caudal to the bladder (yellow asterisk). PSMA: prostate-specific membrane antigen; PET/CT: positron emission tomography/computed tomography; SV: seminal vesicle.

In June 2024, prostate-specific membrane antigen (PSMA) positron emission tomography/computed tomography (PET/CT) (Fig. 2) demonstrated PSMA-avid foci in the anterior midline apex, left lateral margin of the prostate, and base of the right SV. The perirectal cystic lesion showed only low-level PSMA uptake in its wall. There was no evidence of nodal or osseous metastases. Mild left hydronephrosis and hydroureter were noted, with the point of obstruction near the left SV.

Two months later, the patient underwent an extensive, technically challenging robotic-assisted radical prostatectomy with bilateral pelvic lymph node dissection, excision of the large left SV mass, primary rectal repair with omental interposition, and left ureteral reimplantation. The operation was prolonged due to the tumor’s dense adherence to the anterior rectal wall, requiring partial resection and primary repair. The left SV was completely replaced by tumor, with significant adhesions to both the rectum and left ureter. The ureter was transected and reimplanted, and an omental flap was mobilized to reinforce the rectal repair. Colorectal surgery was consulted intraoperatively; although the dissection was complex, no frank rectal breach was identified. The prostate and SVs were ultimately removed *en bloc*.

**Figure 3:**
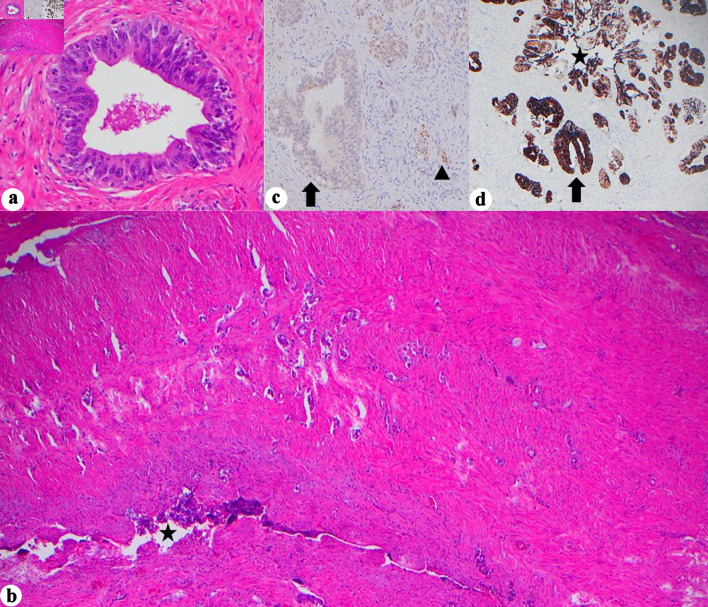
Histopathologic and immunohistochemical features of seminal vesicle involvement. (a) Marked cellular atypia arising in continuity with SV lumen, assumed to be *in situ* component, (H&E, × 400). (b) Glandular infiltration into SV wall (H&E, × 40, star = SV lumen). (c) PAX8 negativity in the *in situ* component but retained in normal SV epithelium (× 400, arrow = *in situ* glands, arrowhead = normal SV). (d) CK7 positivity in the *in situ* component and normal SV glands (× 40, star = SV lumen, arrow = *in situ* glands). H&E: hematoxylin and eosin stain; SV: seminal vesicle; CK: cytokeratin; PAX8: paired box gene 8.

As expected, histologic examination confirmed prostatic acinar adenocarcinoma (Gleason 3 + 4 = 7, grade group 2). The mass was encasing the prostate grossly; however, microscopically it was distinct from the prostatic stroma and was confirmed not to be of prostatic origin by negative NKX3.1. The tumor extensively replaced the soft tissue and showed mostly rhabdoid morphology with some glandular and cystic areas, with focal positive margin. Given the unusual situation, a wide array of immunohistochemical stains were performed, and each component had a different staining pattern. The rhabdoid component was positive for SATB2, focal CAM5.2, and patchy calretinin and negative for PAX8, GATA3, CK7, AE1/3, CK5/6, ERG, SALL4, and p40. The cystic component was positive for p40, HMWK, CAM5.2 (patchy), AE1/3, CK7, calretinin (patchy), CK5/6, and focal GATA3 and was negative for SATB2. INI1 was retained, and both components were negative for chromogranin, synaptophysin, WT1, desmin, SOX10, CD45, MelanA, TTF1, CDX2, and CK20. The key to the diagnosis was in the glandular component, which showed markedly atypical cells in what appeared to be an *in situ* component (Fig. 3a), along with associated infiltrative small glands into SV wall (Fig. 3b). This in situ-appearing glandular component arose in direct continuity with the SV lumen and epithelium, providing morphologic evidence of a primary SV origin rather than secondary involvement from adjacent pelvic organs. In normal SV, PAX8 and CK7 are typically positive; however, in our case, PAX8 was negative in both the *in situ* (Fig. 3c), and invasive glandular component. Given the CK7 positivity (both *in situ* (Fig. 3d), and invasive glandular components) and the apparent *in situ* component, this led to the conclusion of a primary SV tumor.

**Figure 4:**
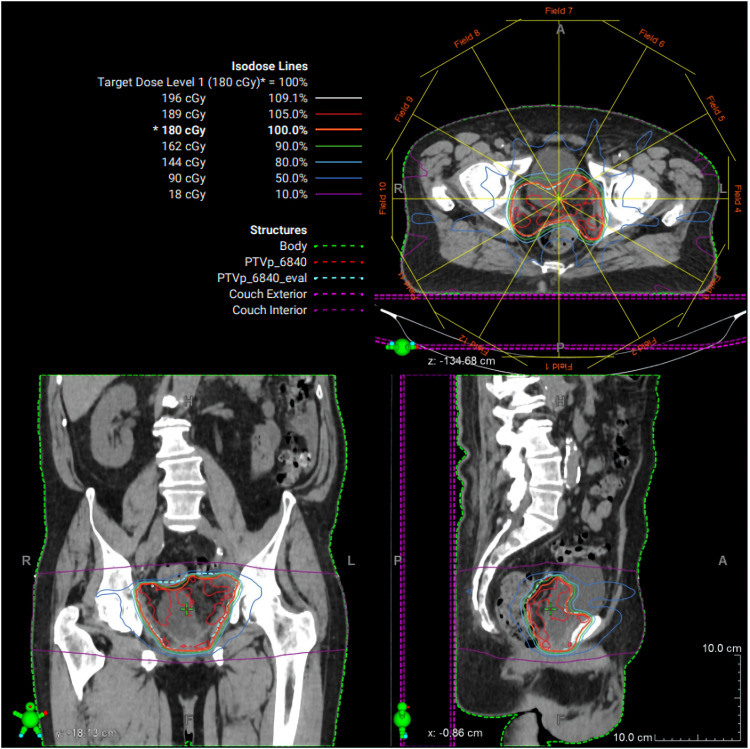
Radiation therapy treatment plan. Representative intensity-modulated radiation therapy (IMRT) plan demonstrating axial, coronal, and sagittal dose distributions for the pelvic target volume. Isodose lines represent percentage dose levels relative to the prescribed 180 cGy fraction. The planning target volume is shown in green, with color-coded isodose curves illustrating conformal coverage of the target and sparing of surrounding normal tissues.

**Figure 5:**
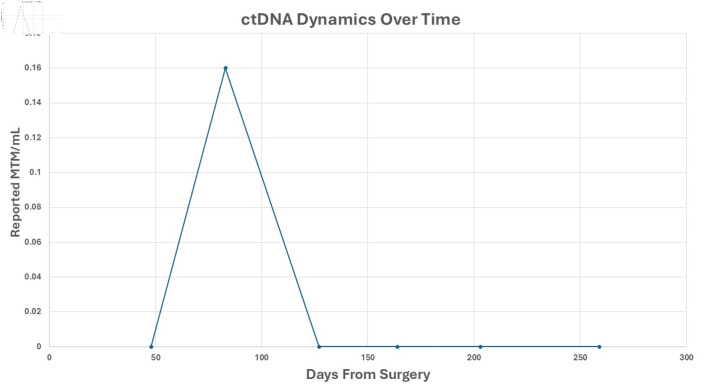
ctDNA dynamics over time. Reported ctDNA levels were measured as MTM/mL, following surgical resection. Each point represents a postoperative plasma sample collected at the indicated time (days from surgery). A transient ctDNA peak was observed at approximately 70 days, followed by sustained clearance thereafter. ctDNA: circulating tumor DNA; MTM/mL: mean tumor molecules per milliliter.

Postoperatively, PSA remained undetectable; however, the extensive SV mass prompted additional biomarker surveillance, and ctDNA (Signatera^®^, Natera, Inc., Austin, TX, USA) testing was elected. Two months later, ctDNA was positive for minimal residual disease. Adjuvant radiation therapy (RT) to the postoperative bed was administered based on high-risk features of the PSVA, including tumor extent and margin status, using intensity modulated RT to 68.4 Gy in 38 fractions with daily image guidance (Fig. 4). Given undetectable PSA and lack of high-risk pathologic features of the synchronous prostate cancer, androgen deprivation therapy was not delivered concurrently with RT. Following RT, the patient’s ctDNA converted to negative (Fig. 5). PSA remained undetectable throughout the postoperative course. Follow-up CT scans in July and October 2025 showed no evidence of recurrence.

## Discussion

PSVA remains one of the least frequently encountered urologic malignancies, with diagnostic and therapeutic challenges amplified by its anatomic location and lack of pathognomonic clinical features. In the largest review to date, most cases occurred in men aged 50–70 years, with a median tumor size of 5–6 cm and frequent local invasion at diagnosis [3]. Our patient’s presentation with a large (7.4 cm) cystic pelvic mass replacing the left SV is consistent with these findings but is unusual in its dedifferentiated morphology and synchronous prostate adenocarcinoma. Accurate diagnosis of PSVA relies on histopathologic and immunohistochemical assessment. Typical profiles include CK7 positivity, CK20 negativity, and variable expressions of CA125 and PAX8 [12]. In our case, the different histology components complicated interpretation, and the initial cyst fluid cytology suggested high-grade urothelial carcinoma, likely sampling from the cystic component, illustrating the potential for misdiagnosis.

Management of PSVA typically involves radical surgery as part of a multimodal approach, often combined with hormone therapy, radiotherapy, and/or chemotherapy [13]. While local excision has been attempted in select cases, complete resection is frequently limited by tumor adherence to surrounding pelvic structures [3]. Radical operations described in the literature include radical prostatectomy, radical cysto prostatectomy, and total pelvic exenteration, often with pelvic lymphadenectomy. The best outcomes appear to occur when radical surgery with negative margins is followed by adjuvant therapy [8, 14-16]. Hormonal manipulation using estrogens [17], bilateral orchiectomy, or androgen deprivation has been applied for local control and metastatic disease [3]. Radiotherapy is used both for adjuvant treatment of residual disease and for nodal metastases, while chemotherapy regimens such as docetaxel–cisplatin, cisplatin–5FU, or oxaliplatin-based combinations have been employed for distant metastases [13]. Despite aggressive multimodal treatment, PSVA carries a poor prognosis, with frequent recurrence and progression; approximately 95% of patients die within 3 years, largely due to delayed diagnosis [3].

Our patient underwent complete surgical excision with focal positive margin for PSVA and negative margin for prostatic adenocarcinoma, followed by adjuvant external beam radiation, consistent with the most frequently reported curative-intent strategy.

While detectable ctDNA may reflect either residual local disease or occult micrometastatic spread, its role in directing treatment selection in PSVA remains undefined. In this case, the decision not to pursue systemic chemotherapy was based on the absence of radiographic evidence of distant disease at the time of treatment planning and the prioritization of achieving durable local control for a high-risk, margin-positive SV malignancy. The subsequent normalization of ctDNA following completion of radiotherapy was reassuring in the post-treatment setting and supported continued surveillance rather than escalation to systemic therapy, in conjunction with persistently negative systemic imaging.

A distinguishing aspect of this case is the use of ctDNA for postoperative molecular surveillance. While ctDNA is increasingly applied in other solid tumors [18], its use in PSVA has not been previously reported. The ctDNA assay used in this case was tumor-informed and designed to track patient-specific somatic variants longitudinally in plasma. Such assays are primarily used for detection of molecular residual disease and assessment of treatment response. However, ctDNA detection depends on tumor shedding and assay sensitivity, does not localize disease, and cannot distinguish residual local disease from occult distant micrometastases. Accordingly, ctDNA findings were interpreted as complementary surveillance information rather than a determinant of treatment selection.

In our patient, the indication for adjuvant radiotherapy in the setting of combined prostate adenocarcinoma and PSVA was driven by the SV malignancy, as there were no high-risk features for biochemical recurrence from the prostate cancer, and PSA was undetectable. The presence of a positive SV margin and detectable postoperative ctDNA supported concern for residual disease related to PSVA. Following adjuvant radiation, ctDNA converted to negative, and the patient has remained disease-free for nearly 1 year, emphasizing a favorable molecular response. Given the historically high local recurrence rates in PSVA, ctDNA may represent a useful adjunct for postoperative surveillance alongside conventional imaging and serum-based follow-up.

### Conclusions

PSVA is a rare and diagnostically challenging malignancy that often presents with advanced local diseases. This case underscores the importance of comprehensive imaging, immunohistochemical profiling, and a high index of suspicion when evaluating SV masses. Multimodal management, including complete surgical excision, adjuvant radiation, and close surveillance, can achieve durable disease control, even in high-grade or dedifferentiated variants. Our report is the first to document molecular clearance using ctDNA in this disease, highlighting its potential utility as a sensitive surveillance tool in rare urologic cancers.

### Learning points

PSVA is an exceptionally rare malignancy and should be considered in the differential diagnosis of cystic or solid SV masses, particularly when imaging features are atypical or disproportionate to prostate findings. Immunohistochemistry is essential for distinguishing PSVA from other pelvic malignancies, especially in cases with dedifferentiated morphology or synchronous genitourinary cancers. Aggressive multimodal management, including complete surgical excision and adjuvant radiotherapy, can achieve durable local disease control. ctDNA may serve as an adjunctive tool for postoperative molecular surveillance in rare urologic malignancies that lack reliable serum biomarkers.

## Data Availability

The data that support the findings of this study are available on request from the corresponding author.

## Abbreviations

PSVA: primary seminal vesicle adenocarcinoma
T3b: tumor stage T3b (extracapsular extension into SVs)
LUTS: lower urinary tract symptoms
MRI: magnetic resonance imaging
PSA: prostate-specific antigen
ctDNA: circulating tumor DNA
PI-RADS: Prostate Imaging–Reporting and Data System
PSMA: prostate-specific membrane antigen
PET/CT: positron emission tomography/computed tomography
CT: computed tomography
CK: cytokeratin
CA-125: cancer antigen 125
PAX8: paired box gene 8
